# Pretomanid vs delamanid in a bedaquiline-linezolid regimen: efficacy in a high-burden tuberculosis mouse model

**DOI:** 10.1128/aac.01953-25

**Published:** 2026-04-29

**Authors:** Jason E. Cummings, Lisa K. Woolhiser, Vincent Guglielmi, Ashely Romano, Samantha Pauly, Matt Zimmerman, Firat Kaya, Nicholas D. Walter, Gregory T. Robertson, Richard A. Slayden

**Affiliations:** 1 Mycobacteria Research Laboratories, Microbiology, Immunology and Pathology, Colorado State University 164598 , Fort Collins, Colorado, USA; 2 Consortium for Applied Microbial Metrics, Aurora, Colorado, USA; 3 Division of Pulmonary Sciences and Critical Care Medicine, University of Colorado Anschutz Medical Campus 199566 , Aurora, Colorado, USA; 4 Center for Discovery and Innovation, Nutley, New Jersey, USA; 5 Rocky Mountain Regional VA Medical Center 19982 , Aurora, Colorado, USA; Queen Mary University of London, London, United Kingdom

**Keywords:** high-burden aerosol BALB/c model, high burden, tuberculosis

## Abstract

The bedaquiline, pretomanid, and linezolid (BPaL) regimen has shown significant efficacy in treating patients with multidrug-resistant and extensively drug-resistant tuberculosis. This study assessed whether delamanid, a closely related nitroimidazole, could replace pretomanid in the BPaL regimen by directly comparing the efficacy of bedaquiline, delamanid, and linezolid (BDL) and BPaL regimens in a high-burden preclinical *Mycobacterium tuberculosis* mouse model. Treatment with pretomanid and delamanid monotherapies and dual therapy with bedaquiline–linezolid was also conducted for comparison. Mice were treated for 2, 4, or 8 weeks, and treatment efficacy was assessed by reductions in lung colony-forming units (CFU), effect size, ribosomal RNA synthesis ratio (RS ratio), and histopathological changes. BPaL showed greater early reduction than BDL, with better CFU reduction kinetics on day 12 (3.96 log_10_ CFU reduction for BPaL vs 2.89 log_10_ CFU reduction for BDL, *P* < 0.001) and a lower RS ratio. By day 26, both BPaL and BDL reduced bacterial counts by nearly 6 log_10_ CFU, and by day 54 of treatment, lung CFU levels in both groups had fallen below detectable levels. Histopathological analysis revealed slightly improved lung inflammation during early treatment in BPaL-treated mice compared to BDL. Plasma-drug levels were measured, confirming that exposures between regimens over the 24-h dosing interval were comparable and not significantly different. These results suggest that although pretomanid remains the preferred component of the BPaL regimen, delamanid could be a viable alternative if there are clinical reasons to substitute, including drug resistance or if pretomanid is unavailable or poorly tolerated.

## INTRODUCTION

Tuberculosis (TB), particularly drug-resistant forms, has historically required treatment durations of 18–24 months with complex multidrug regimens that carry significant toxicities, including hepatotoxicity, myelosuppression, and nephrotoxicity (1–4). These prolonged treatments contribute to poor patient adherence, treatment failure, and the development of additional drug resistance (5). The development of novel nitroimidazole agents such as pretomanid and delamanid represents a substantial improvement in TB management, enabling combination regimens that may shorten treatment to 6 months or less while reducing overall drug burden (6).

Pretomanid and delamanid can each be combined with bedaquiline (B) and linezolid (L) to create the bedaquiline, pretomanid, and linezolid (BPaL) and bedaquiline, delamanid, and linezolid (BDL) regimens, respectively (6). The BPaL regimen has demonstrated robust efficacy in pivotal trials, such as the Nix-TB trial, which achieved >90% success in treating extensively drug-resistant (XDR)-TB or treatment-intolerant multidrug-resistant (MDR)-TB, and the ZeNix trial, which established that linezolid dose reductions preserve efficacy while improving tolerability (7, 8). Evidence largely derives from smaller observational studies and pharmacological investigations, including the DELIBERATE trial, which confirmed the acceptable tolerability of the bedaquiline–delamanid combination (9). Despite sharing the F420-dependent activation pathway and overlapping targets including DprE2 and NAD adduct formation, pretomanid and delamanid exhibit distinct pharmacokinetic profiles, bactericidal kinetics, and resistance mutation patterns, differences that may have meaningful consequences for regimen-level performance (10, 11). Clinical resistance to bedaquiline has been documented in both regimen contexts, underscoring the importance of a companion drug with adequate potency to protect the bedaquiline anchor from the risks of functional monotherapy (10, 12–14).

**Fig 1:**
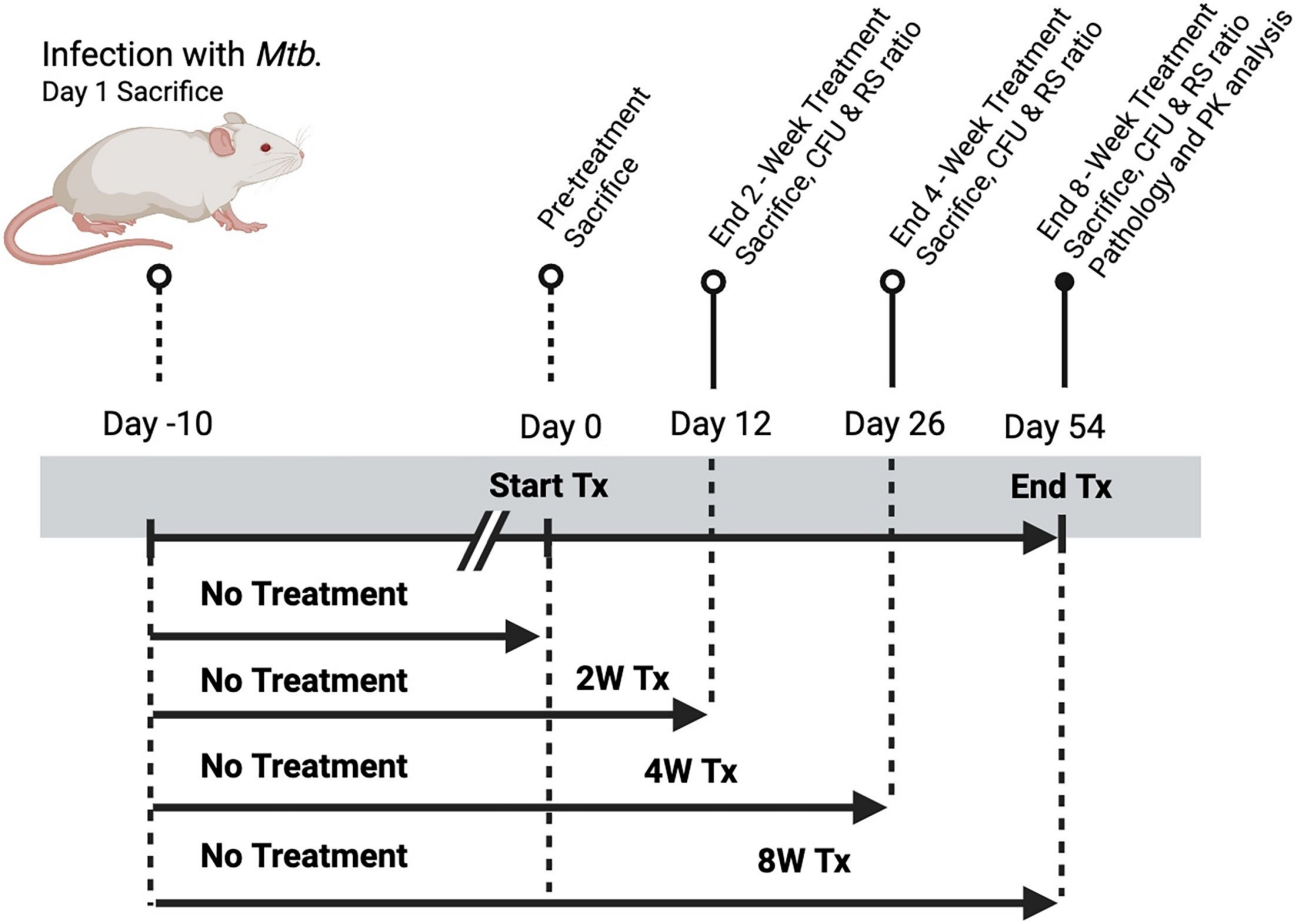
Experimental design and treatment schedule for *Mycobacterium tuberculosis* infection and therapeutic intervention. BALB/c mice were infected with *M. tuberculosis* via the aerosol route on day 10. One group of mice was sacrificed post-infection to assess baseline bacterial burden. The remaining animals were left untreated until day 0, when treatment (Tx) was initiated. Treatment durations included 2, 4, and 8 weeks of drug administration, ending on days 12, 26, and 54, respectively. Sacrifices were conducted at pretreatment (day 0) and post-treatment time points (days 12, 26, and 54) for quantification of colony-forming units (CFU) and rRNA synthesis ratio. On day 54, additional analyses were performed, including pathology and pharmacokinetic assessments.

To address the limited comparative data between the nitroimidazoles pretomanid and delamanid, we evaluated whether substituting delamanid for pretomanid within a bedaquiline–linezolid (BL) backbone preserves regimen-level efficacy. Using a high-burden murine model of pulmonary tuberculosis, we directly compared BPaL and BDL across multiple treatment intervals, assessing lung bacterial burden (colony-forming units [CFU]), ribosomal RNA (rRNA) synthesis ratio (RS ratio), histopathology, and terminal serum drug exposures (Fig. 1). Our findings demonstrate that although pretomanid mediates more rapid early bacterial clearance, delamanid achieves comparable sterilizing activity and resolution of lung pathology by the end of treatment. These results define the pharmacodynamic consequences of nitroimidazole substitution in bedaquiline–linezolid–based regimens and inform the rational integration of analogs into MDR-TB therapy.

## MATERIALS AND METHODS

### High-burden mouse model

Female BALB/c mice (6–8 weeks old; Jackson Laboratories, Sacramento, CA, USA) were received and housed in the animal biosafety level 3 for 1 week before infection. The study employed a high-dose aerosol respiratory infection model using the *Mycobacterium tuberculosis* Erdman strain. The model simulates severe TB infection, with high bacterial burdens in the lungs consistent with severe human MDR/XDR TB. *Mycobacterium tuberculosis* cultures were prepared and grown to mid-log phase in 7H9-glycerol-albumin, dextrose, and catalase (ADC) media containing 0.05% Tween 80. On the day of infection, cultures were adjusted to an optical density at 600 nm (OD_600_) of 0.8 and aerosolized using a Glas-Col Inhalation Exposure System. Mice were exposed to the aerosol for 40 min, resulting in a deposition of approximately 4.29 ± 0.02 log_10_ CFU/lung. Three mice were euthanized 24 h post-infection to confirm bacterial deposition; lungs were harvested/homogenized and cultured on solid media. Treatment commenced on day 11 post-infection, following a 10-day period for infection establishment, and 10 mice were sacrificed. Their lungs were harvested to determine the baseline bacterial load (CFU) and to perform histopathology measurements at the start of treatment. Mice were weighed twice weekly and observed daily for clinical signs of morbidity (e.g., lethargy, ruffled fur, and respiratory distress) throughout the study. Body weight and general health were monitored to assess treatment tolerance and adverse events, and humane endpoints were strictly applied. All animal procedures complied with guidelines established by the Animal Welfare Act and approved by the Institutional Animal Care and Use Committee at Colorado State University. Humane endpoints were strictly adhered to, and mice exhibiting signs of morbidity were euthanized by CO_2_ asphyxiation, confirmed by cervical dislocation in accordance with American Veterinary Medical Association guidelines.

### Treatments

Mice were block-randomized into six groups according to the assigned treatment regimens. The untreated control group (Group 1) received no drug therapy and served as a baseline for comparing the efficacy of the other treatments (sacrificed at the start of treatment). Group 2 received pretomanid (Pa) at a dose of 100 mg/kg. Group 3 received delamanid (D) at a dose of 6 mg/kg. Group 4 received a combination of bedaquiline (B) at 25 mg/kg and linezolid (L) at 100 mg/kg. Group 5 received the standard BPaL regimen: bedaquiline (25 mg/kg), pretomanid (100 mg/kg), and linezolid (100 mg/kg). Finally, Group 6 received the BDL regimen, substituting delamanid (6 mg/kg) for pretomanid and combining it with bedaquiline (25 mg/kg) and linezolid (100 mg/kg). All treatments were administered once daily, 5 days per week, at a volume of 0.2 mL by oral gavage. The treatments were administered 5 of 7 days per week over 8 weeks. Bedaquiline and pretomanid were prepared at twice the concentrations and mixed immediately before oral administration. The other drug solutions were prepared in weekly batches, stored at 4°C, and allowed to reach room temperature before administration.

### Bacterial burden and CFU determinations

At days 12, 26, and 54, groups of mice (*n* = 10 per group) were sacrificed, and their lung tissues were harvested for CFU enumeration. Lungs were aseptically removed, homogenized in 1× phosphate-buffered saline + 10% bovine serum albumin (BSA), and serially diluted for plating on Middlebrook 7H11 agar plates supplemented with 10% oleic acid, albumin, dextrose, and catalase (OADC) and 0.4% wt/vol charcoal. The BSA and charcoal were included to mitigate drug carryover from treated lung tissue. CFUs were counted after 35 days of incubation at 37°C, and the bacterial burden was expressed as log_10_ CFU per lung.

### RS ratio determination

RS ratio is a pharmacodynamic marker of ongoing *Mycobacterium tuberculosis* rRNA synthesis. As previously described, the RS ratio measures the amount of unstable precursor rRNA (pre-rRNA) spacer sequence relative to stable mature rRNA via digital PCR (15, 16). The RS ratio provides additional information on the effectiveness of treatment on pathogen status and transcriptional activity, distinct from and complementary to CFU burden measures, and has previously been shown to indicate the sterilizing activity of regimens in the BALB/c mouse.

### Statistical analysis

All data were expressed as mean ± standard error of the mean. Comparisons between treatment groups were made using one-way analysis of variance (ANOVA), followed by Tukey’s multiple comparisons test. A *P*-value of less than 0.05 was considered statistically significant. Bacterial burden reductions (log_10_ CFU) and RS ratio values were compared at each time point across treatment groups. Cohen’s d was calculated to provide standardized effect size estimates across regimens and time points, as previously described (17, 18).

### Mass spectrometry analysis of drug levels in serum

Serum drug concentrations were quantified using liquid chromatography–tandem mass spectrometry (LC-MS/MS) to assess the pharmacokinetic profiles of each drug within the regimens (11, 19, 20). Blood samples were collected using the submandibular collection method from two mice per group at 1 h post-treatment delivery (*C*_max_) and 24 h post-treatment delivery (trough) during week 7 of treatment. Serum samples were processed and analyzed for bedaquiline, pretomanid, delamanid, and linezolid concentrations. Given that sampling was limited to a single treatment week, two post-dose time points, and *n* = 2 animals per group, these measurements were intended to verify achieved systemic exposure rather than to establish steady-state pharmacokinetic parameters or to support formal exposure–response comparisons between regimens.

### Histopathological analysis

At the start of treatment (baseline, day 0) and at day 54, lung tissues from representative mice (*n* = 4 per group) treated with either BPaL or BDL were fixed in 10% neutral-buffered formalin, embedded in paraffin, sectioned, and stained with hematoxylin and eosin. A board-certified pathologist blinded to the treatment groups assessed histopathological changes in lung tissue, including granuloma formation, necrosis, inflammation, and tissue damage. Lesion scoring was used to compare the severity of lung damage between treatment groups.

## RESULTS

### Comparative efficacy and bactericidal kinetics of pretomanid versus delamanid within bedaquiline–linezolid regimens

**Fig 2:**
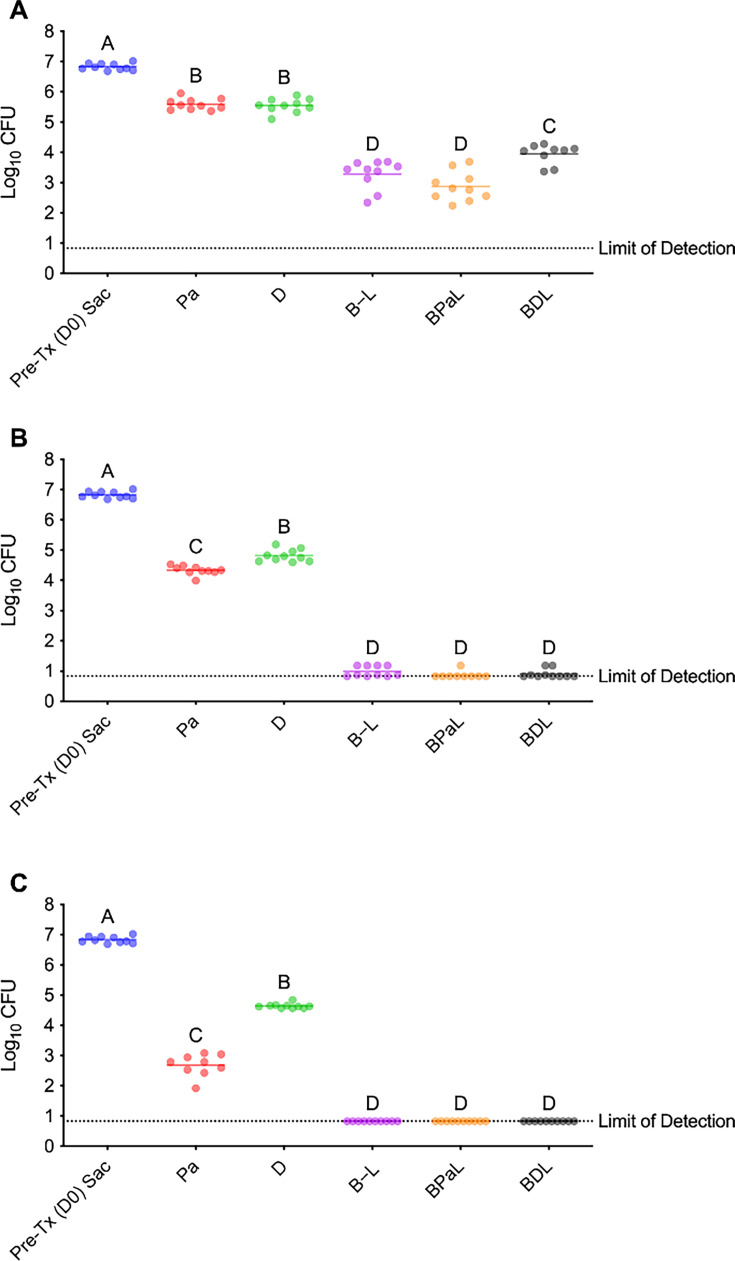
Bacterial burden during treatment. Colony-forming unit (CFU) counts from lungs after treatment of 5 of 7 days per week with indicated drug regimens at day 12 (**A**), day 26 (**B**), and day 54 (**C**). Mice were infected 11 days before the start of treatment (D0) by high-dose aerosol with *Mycobacterium tuberculosis* Erdman. CFU data points represent log_10_ CFU from individual mice. The solid horizontal line indicates the group mean. Dashed lines represent the lower limits of detection. By day 12, the bedaquiline, pretomanid, and linezolid (BPaL) regimen resulted in significantly lower CFU than bedaquiline, delamanid, and linezolid (BDL) (*P* < 0.001). By day 54 (panel C), all combination regimens achieved CFU counts below detection (~0.83 log_10_ CFU, dashed line). Statistical comparisons between groups are summarized using compact letter display: groups sharing a letter are not significantly different (*P* ≥ 0.05). In contrast, those with no letters in common are significantly different (*P* < 0.05).

The primary objective of this study was to determine whether delamanid could effectively substitute for pretomanid in the bedaquiline–linezolid backbone by comparing the bactericidal kinetics and overall efficacy of the BPaL and BDL regimens in a high-burden mouse model of tuberculosis. To contextualize these comparisons, combination regimens (BPaL, BDL, and BL) and monotherapies (Pa and D) were evaluated for their ability to reduce bacterial burden, as measured by colony-forming units (CFUs) in lung tissue at multiple intervals. As expected, multidrug regimens significantly outperformed monotherapies in reducing bacterial counts. By day 12, combination treatments generally showed improved killing kinetics, achieving reductions in lung burdens of 3.96 log_10_ for BPaL, 3.55 log_10_ for BL, and 2.88 log_10_ for BDL, compared to single-drug treatments with Pa and D. BPaL displayed significantly superior killing kinetics over BDL during the first 2 weeks of treatment, with nearly a 1 log_10_ greater reduction in lung CFUs at this point (*P* < 0.001; Fig. 2A). By day 26, all three multidrug regimens resulted in bacterial levels that were nearly undetectable, with reductions of 5.96 log_10_ CFU (BPaL), 5.92 log_10_ CFU (BDL), and 5.85 log_10_ CFU (BL) (Fig. 2B). Differences among multidrug regimens were minimal at this time point and did not reach statistical significance. By day 54, all three regimens reduced the bacterial load below the detection limit (<0.83 log_10_ CFU), resulting in an overall reduction of about 6 log_10_ CFU from the initial pretreatment lung burden of 6.83 log_10_ CFU. Single-drug therapies had slower bacterial clearance compared to multidrug regimens. Pa and D alone reduced bacterial loads by 1.24 log_10_ CFU (*P* < 0.001) and 1.28 log_10_ CFU (*P* < 0.001) by day 12. The bacterial burden reduction with Pa persisted through day 26 (2.49 log_10_ CFU, *P* < 0.001) and day 54 (4.15 log_10_ CFU, *P* < 0.001). Conversely, D treatment lowered bacterial load by 2.01 log_10_ CFU (*P* < 0.001) at day 26 but did not significantly reduce bacterial levels from day 26 to day 54 (2.19 log_10_ CFU, *P* = 0.9588). Table S1 (Table S1) presents the complete statistical data for bacterial burden.

We further analyzed bacterial burden reduction using Cohen’s *d* to evaluate treatment effectiveness beyond ANOVA statistical significance. Cohen’s *d* provides a standardized measure of effect size, enabling comparisons across different regimens and time points (17, 18). Effect sizes were divided into four categories based on quartile thresholds: minimal effect (*d* < 3.38), moderate effect (3.38–7.57), large effect (7.58–25.02), and very large effect (*d* > 25.02). By day 12, all treatments showed large to very large effect sizes compared to pretreatment controls, with BPaL and BDL displaying the strongest effects (Table S1). Pa and D monotherapies initially showed weaker effects, but effect sizes increased by day 54. The combination therapies (BPaL, BDL, and BL) achieved much faster bacterial clearance than single-drug regimens, as evidenced by consistently large to very large effect sizes. Notably, replacing Pa with D in the BPaL regimen (i.e., BDL) did not result in a significant difference in effect size, indicating that both regimens were similarly efficacious.

### BPaL achieved superior suppression of the RS ratio compared to BDL and BL

**Fig 3:**
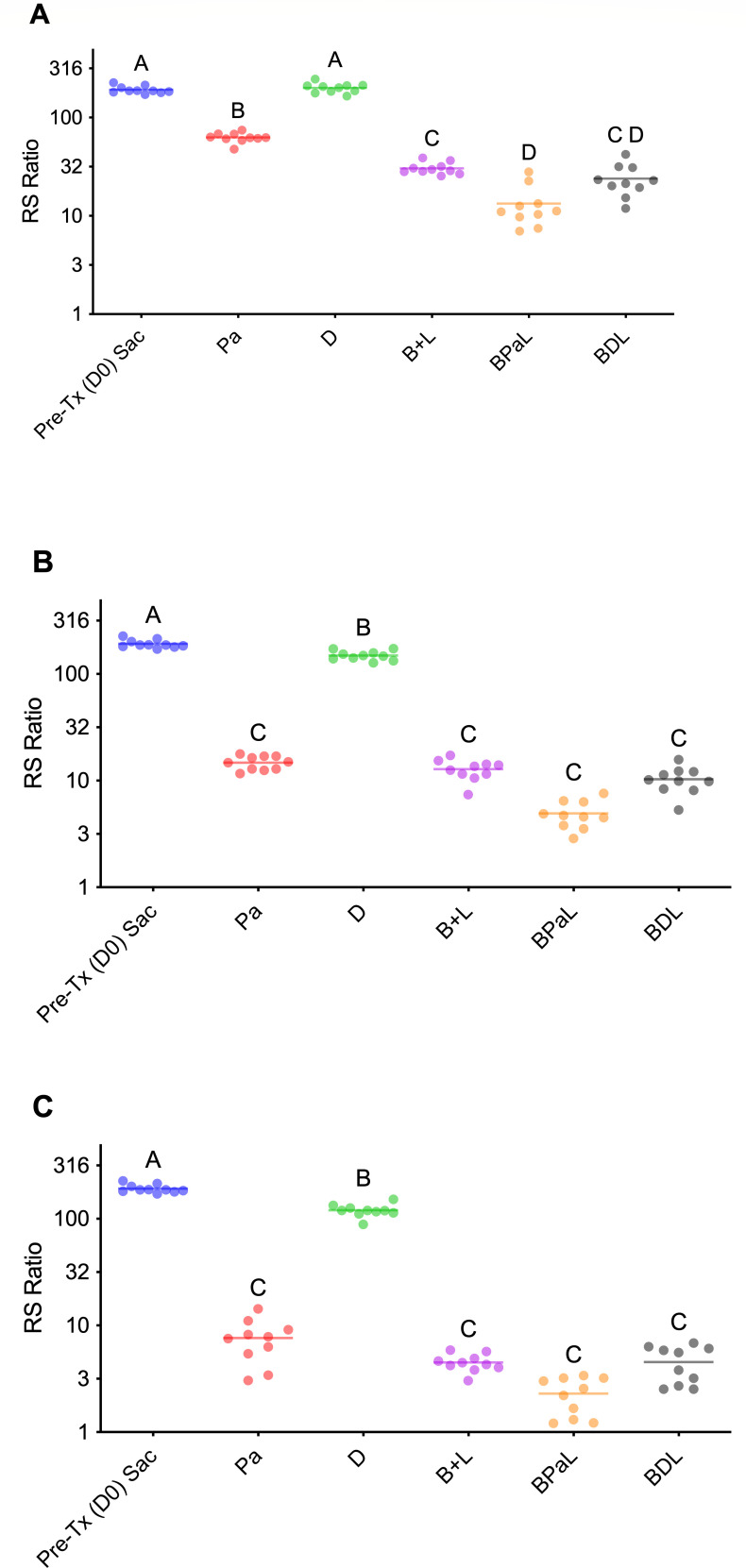
rRNA synthesis ratio (RS ratio) during treatment. RS ratio from lungs after treatment of 5 of 7 days per week with indicated drug regimens at day 12 (**A**), day 26 (**B**), and day 54 (**C**). Mice were infected 11 days before the start of treatment (D0) by high-dose aerosol with *Mycobacterium tuberculosis* Erdman. (**A**) Day 12: bedaquiline, pretomanid, and linezolid (BPaL) significantly lowered RS ratio vs bedaquiline–linezolid (B–L) (*P* < 0.05). (**B**) Day 26: differences between combo regimens narrowed. (**C**) Day 54: all regimens yielded low RS ratios, indicating clearance of transcriptionally active bacteria. RS ratio data points represent values measured from individual mice. The solid horizontal line indicates the group mean. Statistical comparisons between groups are summarized using compact letter display: groups sharing a letter are not significantly different (*P* ≥ 0.05). In contrast, those with no letters in common are significantly different (*P* < 0.05).

The RS ratio, a quantitative and sensitive measure of bacterial transcriptional activity, was used to further assess the effects of the BPaL, BDL, and BL treatment regimens on *M. tuberculosis* transcription. A lower RS ratio indicates reduced rRNA synthesis. Statistical data for the RS ratio are available in Table S2 (Table S2). On day 12, the RS ratio for the BPaL regimen was 13, significantly lower than the RS ratio of 30 for BL (*P* < 0.05), indicating more effective inhibition of rRNA synthesis than the two-drug regimen. However, BPaL was not significantly different from BDL, which had an RS ratio of 24 (*P* = 0.45) at this early time point. Similarly, the RS ratio for BDL was not significantly different from BL (*P* = 0.86). Single-drug treatment with pretomanid (RS ratio of 63) showed a modest improvement, whereas delamanid (RS ratio of 200) did not reduce the RS ratio by day 12 (pretreatment RS ratio = 192). At all time points, pretomanid was more effective at lowering the RS ratio than delamanid, although both were less effective than the combination regimens and showed significantly different effects (Fig. 3A). At day 26, the RS ratio pattern among BPaL, BDL, and BL was similar to that observed at day 12, with combination regimens showing similar RS ratio activity for BL (13.0) and BDL (10.0) and a lower RS ratio for the BPaL regimen (5), all of which were not significantly different. (Fig. 3B). This trend continued through day 54, when the combination regimens reduced the RS ratio to two for BPaL and five for BDL and BL, with no statistically significant differences between the combination regimens (Fig. 3C).

### Histopathological outcomes following BPaL and BDL treatment

Histopathological analysis of lung tissues from BPaL- and BDL-treated mice was performed only at the end of the treatment period (day 54), providing context for interpreting structural recovery and the resolution of inflammation relative to the pretreatment state. At treatment initiation, the lungs displayed signs of granulomatous consolidation, necrosis, and inflammatory infiltration, characteristic of a high-burden *M. tuberculosis* infection (17, 18). By day 54, both BPaL- and BDL-treated mice exhibited marked restoration of normal lung architecture and a substantial reduction in inflammation. In BPaL-treated animals, residual lesions were more organized, with macrophage-rich infiltrates and lymphoid aggregation consistent with immune remodeling following rapid bacterial clearance. BDL-treated lungs showed similarly diminished but less organized inflammatory patterns, suggesting a slower resolution trajectory.

### Pretomanid and delamanid achieved systemic exposures within expected ranges during treatment

To confirm systemic drug delivery during the final week of treatment, serum drug concentrations were quantified by LC–MS/MS at week 7, 1 and 24 h after dosing. All administered drugs were detectable at both time points, confirming successful systemic exposure under the dosing conditions used in this study. Mean bedaquiline concentrations across treatment groups were 0.63 ± 0.19 µg/mL at 1 h and 0.68 ± 0.19 µg/mL at 24 h post-dose, consistent with the drugs’ known half-life. Bedaquiline concentrations in the BPaL group were approximately threefold higher than in the BL and BDL groups at the sampled time points; however, these differences were not statistically significant (adjusted *P* = 0.403 and 0.223, respectively). Delamanid concentrations in the D and BDL groups were 0.18 ± 0.09 µg/mL at 1 h and 0.18 ± 0.04 µg/mL at 24 h, demonstrating relatively stable levels across the dosing interval. Pretomanid concentrations in the Pa and BpaL groups were 7.8 ± 1.1 µg/mL at 1 h and 4.9 ± 2.0 µg/mL at 24 h. Linezolid concentrations in the BL, BpaL, and BDL groups were 24.9 ± 4.1 µg/mL at 1 h and declined to 0.04 ± 0.01 µg/mL at 24 h, consistent with its expected pharmacokinetic profile. Observed pretomanid concentrations were at the upper end of reported human peak exposures, whereas delamanid concentrations were within the lower end of the reported clinical range (21, 22). Pairwise *post hoc* comparisons using Šídák’s and Tukey’s multiple-comparison tests (Table S3) found no statistically significant differences in *C*_max_ or 24-h trough concentrations for pretomanid, delamanid, bedaquiline, or linezolid between corresponding regimens (all adjusted *P* > 0.13).

### Treatment tolerance and adverse events

To assess the safety and tolerability of the regimens, the treatment groups were monitored for adverse events and signs of toxicity. All treatment regimens were generally well tolerated by the mice during the 8-week study, with no adverse events or signs of severe toxicity observed. Mice in all groups maintained or gained weight throughout the study, except for transient weight loss in the first 2 weeks of treatment. This was attributed to the onset of clinical disease following the 11 days of infection establishment before treatment initiation. No signs of clinical illness or distress were observed, and no mice required euthanasia due to treatment-related complications.

## DISCUSSION

This study offers a comparative look at the early and long-lasting effectiveness of the bedaquiline, pretomanid, and linezolid (BPaL) and bedaquiline, delamanid, and linezolid (BDL) regimens in a high-burden murine model of tuberculosis. By examining bacterial clearance, effect size, transcriptional suppression, histopathological results, pharmacokinetics, and treatment tolerance, we aimed to determine whether delamanid could effectively replace pretomanid in a potent three-drug regimen for drug-resistant TB. Given the ongoing global challenge of multidrug-resistant (MDR) and extensively drug-resistant (XDR) TB, finding alternative, rifampicin-sparing treatment options remains a pressing clinical need. Our results fill knowledge gaps by offering back-translational evidence on how these nitroimidazole analogs perform when combined with bedaquiline and linezolid, guiding both preclinical benchmarks and possible clinical use.

BPaL consistently demonstrated improved early bactericidal activity compared with BDL. By day 12, representing only 9 days of treatment, BPaL achieved a nearly 1 log_10_ greater reduction in lung CFU, highlighting pretomanid’s potential to accelerate early bacterial clearance. In contrast, BDL exhibited slower killing kinetics than BPaL. Both pretomanid and delamanid demonstrate modest early bactericidal activity when delivered as monotherapy; however, when combined with BL, only pretomanid contributes to early bactericidal activity, as evidenced by greater reductions in bacterial lung burden compared to the two-drug BL regimen. The slightly improved early bactericidal effect of BPaL resulting from Pa may be clinically advantageous by reducing transmission potential, limiting the progression of TB-associated lung pathology, and reducing transmission (4, 23). By day 26, both BPaL and BDL regimens exhibited further and significant reduction, consistent with near-limit-of-detection declines in bacterial lung burden. At this stage, BPaL and BDL reductions approached six logs and remained numerically greater than BL, although performance differences among multidrug regimens had narrowed considerably. By day 54, BPaL, BDL, and BL multidrug regimens reduced lung CFU below the limit of detection, demonstrating equivalent long-term efficacy despite their divergent early-phase kinetics.

These findings were supported by a quantitative effect-size analysis. Cohen’s *d* consistently classified all multidrug regimens within the large to very large efficacy range, confirming their strong activity beyond pretomanid and delamanid single-drug treatments. This sets a significant benchmark for TB treatments, surpassing the traditional Isoniazid-Rifampicin-Pyrazinamide-Ethambutol (HRZE) regimen in the same high-burden model (17, 18). While the early advantage of BPaL was clear in both CFU reductions and the magnitude of effect size, the convergence of regimens by day 54 suggests that delamanid ultimately offers sterilizing efficacy comparable to pretomanid. This delayed bactericidal activity may indicate differences in metabolic activation pathways or pharmacokinetic distribution within the murine host (12, 23). Overall, these results show that although pretomanid is more effective for rapid clearance, delamanid remains a practical substitute, aligning with clinical observations from smaller studies and the DELIBERATE trial, which confirmed the safety of combining bedaquiline and delamanid in MDR-TB patients (17).

The RS ratio provided additional evidence of regimen effectiveness, indicating suppression of ongoing *M. tuberculosis* rRNA synthesis (24). By day 12, BPaL showed a significantly lower RS ratio than BDL or BL, reflecting its greater ability to reduce the transcriptional activity of residual bacilli. Although BDL also lowered the RS ratio, its effect was less significant than BPaL at this early stage, mirroring the delayed bactericidal kinetics observed in CFU data. By day 26, differences between regimens diminished, with all multidrug combinations exhibiting substantial reductions in transcriptional activity. By day 54, RS ratios approached minimal levels across all groups, indicating clearance of transcriptionally active bacilli. Pretomanid consistently outperformed delamanid as a single agent in lowering RS ratio values, consistent with its faster onset of activity. In contrast, delamanid monotherapy did not significantly reduce the RS ratio at day 12, suggesting limited potency in suppressing active bacterial transcription. Importantly, the correlation between RS ratio suppression and CFU reductions supports the idea that pretomanid contributes more to efficacy than delamanid and predicts treatment-shortening activity.

Histopathological analysis offered further insight into how regimens affect TB-related lung damage (Fig. S1). At the start of treatment, mice showed signs of granulomatous lesion formation, necrosis, and inflammation typical of high-burden infection (17, 18). By day 54, both BPaL- and BDL-treated animals showed improved lung structure. BPaL-treated mice showed improved pathology, characterized by resolving, organized inflammatory areas featuring macrophage-rich infiltrates and lymphoid clusters, likely reflecting antigen-driven immune responses associated with rapid bacterial clearance. BDL-treated animals also exhibited less pathology, although their inflammatory infiltrates were less organized. These results suggest a strong link between rapid bacterial clearance and reduced lung damage. Faster reduction in bacterial burden with BPaL may help resolve inflammation sooner and prevent long-term tissue damage. Nevertheless, BDL ultimately offered similar protection against TB-induced lung injury, highlighting its potential to maintain lung function when substituted for pretomanid. This agrees with earlier studies that associate quicker bacterial killing with better clinical outcomes and fewer fibrotic scars in TB patients (25). A limitation of this study is the use of a BALB/c mouse model, which does not develop caseous necrotic lung lesions characteristic of advanced human tuberculosis. Models that recapitulate caseous pathology may reveal additional similarities or distinctions between pretomanid and delamanid with respect to lesion penetration or activity against heterogeneous bacterial populations (24).

Pharmacokinetic analysis at the end of the study confirmed that systemic exposures to pretomanid, delamanid, and linezolid were within the expected ranges for their respective regimens. The observed differences at the endpoint were consistent with prior reports in both murine and human studies (21, 22). Bedaquiline and linezolid exposures were also monitored to confirm that they were similar across regimens. Although bedaquiline concentrations were lower than those usually reported in humans at steady state, this is likely due to species-specific pharmacokinetics and the limited post-dose sampling window used here, which may have missed the delayed *T*_max_ typical of bedaquiline. Notably, the dosing levels used in this study were intentionally higher than human-equivalent exposures to align with historical preclinical efficacy studies published in mouse models (19, 20). Nevertheless, this dosing provides insight into human equivalence and is therefore used to ensure translational continuity with prior data sets while enabling accurate efficacy benchmarking. Collectively, these findings indicate that systemic exposures for pretomanid and delamanid were within expected ranges and broadly comparable across regimens. However, given the limited and confirmatory nature of the pharmacokinetic sampling, these data are not intended to definitively attribute observed efficacy differences to pharmacokinetic differences.

All regimens were well tolerated in this study, with no adverse events or treatment-related morbidity observed. Mice in all groups maintained or gained weight after an initial transient loss attributable to infection, and no animals required euthanasia for treatment-related causes. These findings support the safety of both BPaL and BDL in clinical settings during these treatment periods. From a clinical perspective, tolerability remains a key factor in a regimen’s usefulness. Linezolid is well-known for its dose-limiting toxicities, including myelosuppression and neuropathy (26, 27). Pretomanid, while effective, has been associated with hepatotoxicity and gastrointestinal intolerance in certain patient populations (28). Delamanid, in contrast, has demonstrated a more favorable safety profile, with fewer reports of neurotoxicity and hepatotoxicity; however, concerns regarding QT prolongation persist (29). Our findings suggest that delamanid may be a valuable clinical alternative for patients who cannot tolerate pretomanid, especially in long-term regimens that rely on bedaquiline and linezolid as core agents.

### Conclusion

This study evaluated whether delamanid could effectively substitute for pretomanid in the bedaquiline–linezolid (BPaL) regimen in a high-burden murine tuberculosis model. Both regimens showed potent efficacy, with pretomanid driving faster early bacterial clearance and delamanid achieving comparable sterilizing activity by the end of treatment. Parallel improvements in bacterial burden, RS ratio, and lung pathology, together with consistent pharmacokinetic profiles, confirm that efficacy differences reflect intrinsic drug properties rather than disparities in exposure. Given delamanid’s favorable safety profile, the BDL regimen is a viable clinical alternative to BPaL, particularly for patients unable to tolerate pretomanid. Collectively, these results validate the translational relevance of back-translational models for assessing pharmacophore substitution and highlight the need for continued investigation into relapse prevention, resistance dynamics, and dose optimization for delamanid-containing regimens. Future research should investigate the relapse-prevention potential of bedaquiline-anchored pretomanid and delamanid regimens, especially considering delamanid’s slower onset of bactericidal activity. Additionally, dose optimization could enhance the comparison of regimen performance, as its stable pharmacokinetics suggests opportunities for refinement to fully define the therapeutic role of BDL in TB treatment.

## Data Availability

The data supporting the findings of this study are available from the corresponding author upon reasonable request.

## SUPPLEMENTAL MATERIAL

Fig. S1 aac.01953-25-s0001.tiff Pathology.

Supplemental tables aac.01953-25-s0002.docx Tables S1 to S3.

ASM does not own the copyrights to Supplemental Material that may be linked to, or accessed through, an article. The authors have granted ASM a non-exclusive, world-wide license to publish the Supplemental Material files. Please contact the corresponding author directly for reuse.
