# Supplementary figures and images for: Pretomanid vs delamanid in a bedaquiline-linezolid regimen: efficacy in a high-burden tuberculosis mouse model
Source: Antimicrob Agents Chemother. 2026 Apr 29;70(6):e01953-25. doi: 10.1128/aac.01953-25 (PMC13231923; doi:10.1128/aac.01953-25)

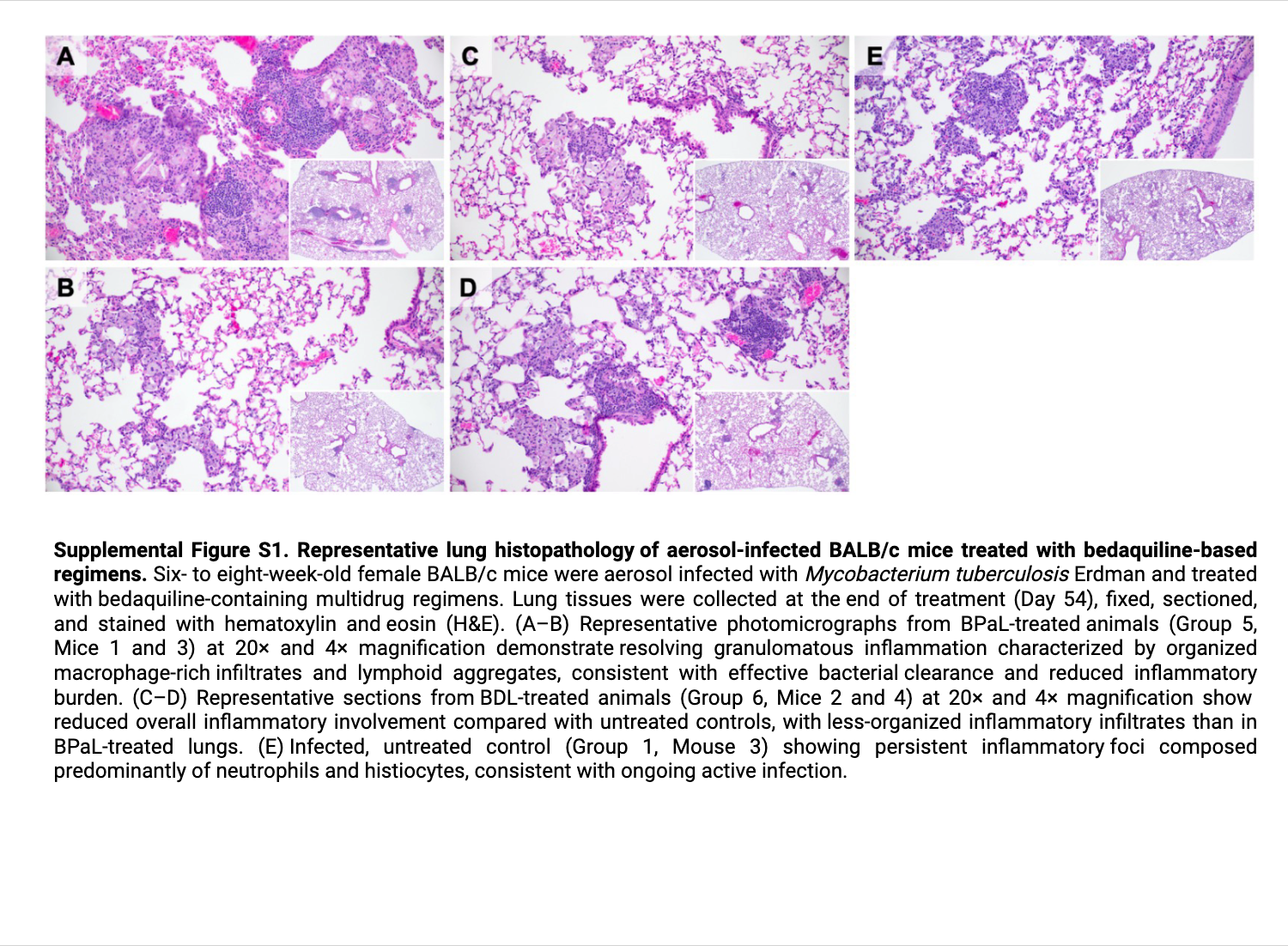

Supplement: Fig. S1 — Pathology. [file aac.01953-25-s0001.tiff]
