# Supplementary material for: Pretomanid vs delamanid in a bedaquiline-linezolid regimen: efficacy in a high-burden tuberculosis mouse model
Source: Antimicrob Agents Chemother. 2026 Apr 29;70(6):e01953-25. doi: 10.1128/aac.01953-25 (PMC13231923; doi:10.1128/aac.01953-25)
Supplement: Supplemental tables — Tables S1 to S3. [file aac.01953-25-s0002.docx]

**Table S1. Bacterial Burden Statistics**

**Table S1.** Mean lung bacterial burden (log₁₀ CFU ± SEM), log₁₀ CFU reductions relative to pretreatment controls, one-way ANOVA results, and Cohen’s *d* effect sizes for pretomanid (Pa), delamanid (D), bedaquiline plus linezolid (BL), BPaL, and BDL regimens at Days 12, 26, and 54 post-treatment initiation. All multidrug regimens demonstrated significant reductions compared with pretreatment controls (p < 0.001).

| **Table S1 Bacterial Burden Statistics.** | | | | | | | |
| --- | --- | --- | --- | --- | --- | --- | --- |
|  | **Day 12** | Pre-Tx Sac | Pa | D | B+L | BPaL | BDL |
|  | Mean Lung Log_10_ CFU | 6.83 | 5.59 | 5.59 | 3.28 | 2.87 | 3.95 |
|  | SEM Lung Log_10_ CFU | 0.04 | 0.06 | 0.07 | 0.15 | 0.15 | 0.11 |
| Compared to Untreated Control | Log_10_ CFU drop | N/A | 1.24 | 1.28 | 3.55 | 3.96 | 2.88 |
|  | ANOVA | N/A | <.001 | <.001 | <.001 | <.001 | <.001 |
|  | Cohen's d | N/A | 8.04 | 7.21 | 10.25 | 11.20 | 12.08 |
|  | **Day 26** | Pre-Tx Sac | Pa | D | B+L | BPaL | BDL |
|  | Mean Lung Log_10_ CFU | 6.83 | 4.34 | 4.82 | 0.98 | 0.87 | 0.91 |
|  | SEM Lung Log_10_ CFU | 0.04 | 0.05 | 0.06 | 0.05 | 0.04 | 0.05 |
| Compared to Untreated Control | Log_10_ CFU drop | N/A | 2.49 | 2.01 | 5.85 | 5.96 | 5.92 |
|  | ANOVA | N/A | <.001 | <.001 | <.001 | <.001 | <.001 |
|  | Cohen's d | N/A | 19.01 | 12.39 | 40.65 | 51.65 | 46.78 |
|  | **Day 54** | Pre-Tx Sac | Pa | D | B+L | BPaL | BDL |
|  | Mean Lung Log_10_ CFU | 6.83 | 2.68 | 4.64 | 0.83 | 0.83 | 0.83 |
|  | SEM Lung Log_10_ CFU | 0.04 | 0.12 | 0.03 | 0.00 | 0.00 | 0.00 |
| Compared to Untreated Control | Log_10_ CFU drop | N/A | 4.15 | 2.19 | 6.00 | 6.00 | 6.00 |
|  | ANOVA | N/A | <.001 | <.001 | <.001 | <.001 | <.001 |
|  | Cohen's d | N/A | 15.79 | 22.97 | 76.75 | 76.75 | 76.75 |

**Table S2. RS Ratio Statistics**

**Table S2.** Mean RS ratio (± SEM), reductions relative to pretreatment controls, and one-way ANOVA results for Pa, D, BL, BPaL, and BDL regimens at Days 12, 26, and 54. RS ratio values reflect relative suppression of *M. tuberculosis* rRNA synthesis during treatment.

| **Table S2 RS Ratio Statistics.** | | | | | | | |
| --- | --- | --- | --- | --- | --- | --- | --- |
|  | **Day 12** | Pre-Tx Sac | Pa | D | B+L | BPaL | BDL |
|  | Mean RS Ratio | 192 | 63 | 200 | 30 | 13 | 24 |
|  | SEM RS Ratio | 5.40 | 2.20 | 7.20 | 1.30 | 2.10 | 2.80 |
| Compared to Untreated Control | RS Ratio drop | N/A | 129 | -8 | 162 | 179 | 168 |
|  | ANOVA | N/A | <.001 | 0.688 | <.001 | <.001 | <.001 |
|  | **Day 26** | Pre-Tx Sac | Pa | D | B+L | BPaL | BDL |
|  | Mean RS Ratio | 192 | 15 | 149 | 13 | 5 | 10 |
|  | SEM RS Ratio | 5.40 | 0.69 | 4.80 | 0.87 | 0.46 | 0.89 |
| Compared to Untreated Control | RS Ratio drop | N/A | 177 | 43 | 179 | 187.1 | 182 |
|  | ANOVA | N/A | <.001 | <.001 | <.001 | <.001 | <.001 |
|  | **Day 54** | Pre-Tx Sac | Pa | D | B+L | BPaL | BDL |
|  | Mean RS Ratio | 192 | 8 | 120 | 5 | 2 | 5 |
|  | SEM RS Ratio | 5.40 | 1.10 | 5.10 | 0.27 | 0.28 | 0.55 |
| Compared to Untreated Control | RS Ratio drop | N/A | 184.4 | 72 | 187.5 | 189.7 | 187.5 |
|  | ANOVA | N/A | <.001 | <.001 | <.001 | <.001 | <.001 |

**Table S3. Serum Drug Concentration Comparisons**

**Table S3.** Pairwise statistical comparisons of serum drug concentrations between regimens at 1 hour and 24 hours post-dose during week 7 of treatment. Mean differences, 95% confidence intervals, adjusted p-values, and test type (Šídák or Tukey multiple-comparison tests) are shown. No adjusted comparisons reached statistical significance (α = 0.05).

| **Table S3 Statistical Comparisons of Serum Drug Concentrations between Regimens (Šídák and Tukey Multiple-Comparison Tests).** | | | | | | | |
| --- | --- | --- | --- | --- | --- | --- | --- |
| **Drug** | **Comparison** | **Time (post-dose)** | **Mean Diff. (ng/mL)** | **95 % CI of Diff.** | **Adj. p Value** | **Test** | **Summary** |
| Pretomanid | Pa vs BPaL | 1 h | 165 | -10 224 to 10 554 | 0.998 | Šídák | ns |
| Pretomanid | Pa vs BPaL | 24 h | -5395 | -15 784 to 4 994 | 0.269 | Šídák | ns |
| Delamanid | D vs BDL | 1 h | -258 | -622 to 106 | 0.133 | Šídák | ns |
| Delamanid | D vs BDL | 24 h | -115 | -479 to 249 | 0.555 | Šídák | ns |
| Bedaquiline | BL vs BPaL | 1 h | -761 | -1 886 to 364 | 0.175 | Tukey | ns |
| Bedaquiline | BL vs BDL | 1 h | 54 | -1 071 to 1 179 | 0.988 | Tukey | ns |
| Bedaquiline | BPaL vs BDL | 1 h | 815 | -310 to 1 940 | 0.145 | Tukey | ns |
| Bedaquiline | BL vs BPaL | 24 h | -510 | -1 635 to 615 | 0.403 | Tukey | ns |
| Bedaquiline | BL vs BDL | 24 h | 181 | -944 to 1 306 | 0.877 | Tukey | ns |
| Bedaquiline | BPaL vs BDL | 24 h | 691 | -434 to 1 816 | 0.223 | Tukey | ns |
| Linezolid | BL vs BPaL | 1 h | 8800 | -16 943 to 34 543 | 0.576 | Tukey | ns |
| Linezolid | BL vs BDL | 1 h | 2950 | -22 793 to 28 693 | 0.935 | Tukey | ns |
| Linezolid | BPaL vs BDL | 1 h | -5850 | -31 593 to 19 893 | 0.774 | Tukey | ns |
| Linezolid | BL vs BPaL | 24 h | -1051 | -26 794 to 24 692 | 0.991 | Tukey | ns |
| Linezolid | BL vs BDL | 24 h | 8 | -25 735 to 25 751 | 1 | Tukey | ns |
| Linezolid | BPaL vs BDL | 24 h | 1059 | -24 684 to 26 802 | 0.991 | Tukey | ns |
| Statistical comparisons were performed using Šídák’s or Tukey’s multiple-comparison tests (α = 0.05). No adjusted p-values reached significance. See Table S3 for group means ± SEM. | | | | | | | |
